# Herpes zoster vaccine effectiveness against herpes zoster and postherpetic neuralgia in New Zealand: a retrospective cohort study

**DOI:** 10.1016/j.lanwpc.2022.100601

**Published:** 2022-09-26

**Authors:** James F. Mbinta, Alex X. Wang, Binh P. Nguyen, Janine Paynter, Prosper Mandela A. Awuni, Russell Pine, Andrew A. Sporle, Colin R. Simpson

**Affiliations:** a School of Health, Wellington Faculty of Health, Victoria University of Wellington, Wellington, New Zealand; b School of Mathematics and Statistics, Wellington Faculty of Engineering, Victoria University of Wellington, Wellington, New Zealand; c Department of General Practice & Primary Healthcare, The University of Auckland, Auckland, New Zealand; d School of Kinesiology and Health Sciences, Laurentian University, Sudbury, Canada; e iNZight Analytics Ltd; Department of Statistics, Faculty of Science, University of Auckland, Auckland, New Zealand; f Usher Institute, The University of Edinburgh, Edinburgh, UK

**Keywords:** Herpes zoster, Postherpetic neuralgia, Varicella zoster virus, Zoster vaccine live, New Zealand, AI diseases, Autoimmune diseases, CVD, Cerebrovascular diseases, COPD, Chronic obstructive pulmonary diseases, CI, Confidence interval, DM, Diabetes mellitus, DHB, District health board, HR, Hazard ratio, Adj HR, Adjusted hazard ratio, ICD-10-AM-iii, International Statistical Classification of Diseases and Related Health Problems-Tenth Revision-Australian Modification, IHD, Ischaemic heart diseases, NZ, New Zealand, NZDep2013, New Zealand Socioeconomic 2013 deprivation index, PPV, Positive predictive value, PHN, Postherpetic neuralgia, RCTs, Randomised control trials, VZV, Varicella zoster virus, ZVL, Zoster vaccine live, HZ, Herpes zoster, MELAA, **Middle Eastern / Latin American / African**

## Abstract

**Background:**

Herpes zoster (HZ) and associated complications cause significant burden to older people. A HZ vaccination programme was introduced in Aotearoa New Zealand in April 2018 with a single dose vaccine for those aged 65 years and a four-year catch up for 66–80 year-olds. This study aimed to assess the ‘real-world’ effectiveness of the zoster vaccine live (ZVL) against HZ and postherpetic neuralgia (PHN).

**Methods:**

We conducted a nationwide retrospective matched cohort study from 1 April 2018 to 1 April 2021 using a linked de-identified patient level Ministry of Health data platform. A Cox proportional hazards model was used to estimate ZVL vaccine effectiveness (VE) against HZ and PHN adjusting for covariates. Multiple outcomes were assessed in the primary (hospitalised HZ and PHN – primary diagnosis) and secondary (hospitalised HZ and PHN: primary and secondary diagnosis, community HZ) analyses. A sub-group analysis was carried out in, adults ≥ 65 years old, immunocompromised adults, Māori, and Pacific populations.

**Findings:**

A total of 824,142 (274,272 vaccinated with ZVL matched with 549,870 unvaccinated) New Zealand residents were included in the study. The matched population was 93.4% immunocompetent, 52.2% female, 80.2% European (level 1 ethnic codes), and 64.5% were 65–74 years old (mean age = 71.1±5.0). Vaccinated versus unvaccinated incidence of hospitalised HZ was 0.16 vs. 0.31/1,000 person-years and 0.03 vs. 0.08/1000 person-years for PHN. In the primary analysis, the adjusted overall VE against hospitalised HZ and hospitalised PHN was 57.8% (95% CI: 41.1–69.8) and 73.7% (95% CI:14.0–92.0) respectively. In adults ≥ 65 years old, the VE against hospitalised HZ was 54.4% (95% CI: 36.0–67.5) and VE against hospitalised PHN was 75·5% (95% CI: 19.9–92.5). In the secondary analysis, the VE against community HZ was 30.0% (95% CI: 25.6–34.5). The ZVL VE against hospitalised HZ for immunocompromised adults was 51.1% (95% CI: 23.1–69.5), and PHN hospitalisation was 67.6% (95% CI: 9.3–88.4). The VE against HZ hospitalisation for Māori was 45.2% (95% CI: −23.2–75.6) and for Pacific Peoples was 52.2% (95% CI: −40.6 –83·7).

**Interpretation:**

ZVL was associated with a reduction in risk of hospitalisation from HZ and PHN in the New Zealand population.

**Funding:**

Wellington Doctoral Scholarship awarded to JFM.

**Research in context**

**Evidence before this study**

Herpes zoster vaccines were first licensed in 2006 with the zoster vaccine live (ZVL) being first approved for use in the USA in 2006. ZVL is available in many high-income countries. New Zealand began a national herpes zoster vaccination programmes using the ZVL vaccine in 2018. We searched Medline (Ovid), Embase (Ovid), Cochrane libraries, CINAHL, ProQuest Central, and Dimensions for post-licensure observational studies that assessed the effectiveness of ZVL against herpes zoster (HZ) and postherpetic neuralgia (PHN) published up to January 2021. We found 23 studies that assessed the effectiveness of the ZVL in adults 50 years and older: 16 were carried out in the United States of America, four in the United Kingdom, one in Canada, one in Australia and one in Sweden. The overall vaccine effectiveness (VE) for ZVL against incident HZ ranged from 33.0% to 65.2%. The VE for preventing PHN ranged from 50.0% to 81.0%. We wished to assess the real-world vaccine effectiveness of ZVL offered to Aotearoa New Zealand residents in a new vaccination programme, which began in April 2018.

**Added value of this study**

This is the first evaluation of the herpes zoster vaccination programme in New Zealand which includes an analysis of Māori, the indigenous people (tāngata whenua) of New Zealand. We found that ZVL was effective in preventing hospitalised HZ (57.8%), hospitalised PHN (73.7%), and non-hospitalised HZ (30.0%) in adults. Amongst immunocompromised adults ZVL reduced the risk of HZ hospitalisation with a VE of 51.1% and PHN hospitalisation was 65.2%. The ZVL VE against HZ hospitalisation in people with chronic obstructive pulmonary disease was 47.6%, diabetes: 38.0%, and ischaemic heart disease: 57.5%. The VE against HZ hospitalisation among the Māori was 45.2% and among the Pacific Peoples was 52.2%.

**Implications for all the available evidence**

Using a national “realworld” data platform, we found that ZVL reduced the risk of HZ hospitalisation, PHN hospitalisation and community HZ. Similar VEs were found among immunocompromised adults.

Alt-text: Unlabelled box

## Introduction

Herpes zoster (HZ) or shingles is a painful dermatomal vesicular disease which, results from the reactivation of latent varicella-zoster virus (VZV) in the nerve ganglia when VZV-specific cell-mediated immunity declines.^1^^,^^2^ The main risk factors of HZ are increasing age and immunosuppression.^3^^,^^4^ Almost one third of people are at risk of developing HZ during their lifetime, and almost half of people aged ≥ 85 years will develop HZ.^1^^,^^2^^,^^5^

In Aotearoa New Zealand (NZ), prior to the introduction of the ZVL vaccine programme (2005–15), HZ incidence was 4.9 per 1000 patient-years. Highest rates of HZ were found amongst adults aged 80–90 years (12.8 per 1000 patient years).^6^^,^^7^

Postherpetic neuralgia (PHN) is the commonest complication after HZ (5% to 30%) and presents with debilitating pain which can persist for an extended period (usually ≥ 90 days).^1^^,^^5^^,^^8^ Herpes zoster ophthalmicus (HZO) occurs when VZV reactivation affects the ophthalmic branch of the trigeminal nerve.^5^ HZO develops in 10–15% of people with HZ. Between 30% and 78% of people with HZO develop eye complications (e.g., keratitis, corneal ulceration, iridocyclitis and blindness).^5^

Although there are numerous therapeutic options, the management of HZ and associated complications is often suboptimal (only 50% of patients experience partial symptom relief).^1^^,^^9^ Given the significant impact of HZ and associated complications on health and quality of life (physical, psychological, functional, and social), and increasing economic burden, prevention through vaccination (especially among older adults with comorbidities) is a priority.^9^, ^10^, ^11^

HZ vaccines can boost cell mediated immunity and prevent VZV reactivation.^1^ The Zoster vaccine live (ZVL) is licenced and marketed in many countries for adults ≥ 50 years old.^12^ The safety, efficacy and effectiveness of this vaccine has been previously demonstrated in clinical trials, but further evidence is needed from national real-world observational studies.^10^^,^^13^, ^14^, ^15^, ^16^ In NZ, the single-dose ZVL (also known as the live-attenuated zoster vaccine) was approved on the 1st of April 2018 for use in adults ≥ 50 years old and is available for no charge, to people aged ≥ 65 years (with a catchup for ages 66–80 years which ran from 2018–2021).^17^ To our knowledge, since the vaccine was introduced, no HZ vaccine effectiveness analysis has been carried out in NZ.^16^ Data are also lacking on how the vaccine performs for indigenous peoples and there are few analyses of vaccine effectiveness (VE) and associated complications performed using whole national population data.

Given the relative paucity of herpes zoster vaccine effectiveness studies using whole national linked datasets such as those available in NZ, the information generated on the effectiveness of the ZVL (including Māori, the indigenous people (tāngata whenua) of New Zealand, immunocompromised individuals, and people with comorbidities) will be vital for informing policymakers, health practitioners and the public of the relative benefits of a HZ vaccination programme which uses the ZVL. Our aim was to evaluate the effectiveness of the zoster vaccine live against herpes zoster in the New Zealand population.

## Methods

### Study design and population

A retrospective matched cohort study was conducted using the New Zealand Ministry of Health national data collections (Table S1, Figures S1-2). The National Health Index (NHI)^17^ - that uniquely identifies every healthcare user resident in NZ and was used (for this study) to link the National Minimum Dataset (NMDS),^18^ a national collection of public and private hospital discharge information (including clinical data, for inpatients and day patients), the National Immunisation Registry (NIR)^19^ which includes data for monitoring immunisation coverage and the progress of immunisation campaigns such as the herpes zoster vaccine programme, the Pharmaceutical Collection,^20^ which is a claim and payment information of government subsidised community dispensed pharmaceuticals, the Cancer Registry,^21^ the Mortality Collection,^22^ and the NZ deprivation index (NZDep13; “an area-based measure of relative socio-economic deprivation”).^23^ Multiple data sets were linked by the master NHI number, which is the unique identifier of a patient. The master NHI number guaranteed the exact matching of the health records for a specific patient.

All adults (born prior to 1972) and vaccinated with ZVL between 1^st^ April 2018 and 31^st^ March 2021 were identified through the NIR and considered to be in the vaccinated cohort. The National Immunisation Register is a computerised information system established in 2005 mainly to capture childhood vaccinations. Reporting adult vaccinations is not required by law, but it is recommended, especially for vaccinations administered to age groups eligible for funded HZ vaccinations. Furthermore, HZ completeness is likely to be high as NIR collects data directly from general practice electronic medical records.^19^

A cohort of NHI numbers of vaccinated patients was created; this forms the vaccinated cohort. For the controls, we used the National Health Index, a reference collection of patients who have interacted with the NZ health system and have an NHI number. The coverage is > 98% of the New Zealand population. We removed the vaccinated cohort from this set, forming the group of people eligible to be a control. For each vaccinated patient, unvaccinated controls were selected by matching on age (year of birth), sex, prioritised ethnicity and NZDep2013 and then two people in this group were randomly selected. The random selection was performed using randomised numbers. Sampling occurred without replacement. In cases where matches were not found, then matching was just performed on age (year of birth), sex, and prioritised ethnicity, not NZDep2013. The process of matching was otherwise the same. Also, after matching with year, sex, and ethnicity (dropping NZDep2013), we did a match on year and sex for those that were not matched.^24^ This included those who did not have a diagnosis of HZ one year before the start of the study. A sub-group analysis was performed to understand the vaccine effectiveness in the Māori and Pacific peoples in NZ.

### Exposure definition and outcome assessment

HZ vaccine was administered to individuals and recorded on the NIR. The eligible population of NZ residents was ∼800,000. Participants were followed from the start of study (1^st^ April 2018) to index date (for vaccinated individuals) or until the health outcome of interest or end of study (31^st^ March 2021). We defined the index date as the vaccination date recorded in the NIR plus thirty days. The start of study was considered the index date for the unvaccinated cohort.

We assessed ZVL VE against hospitalised HZ, community treated HZ and hospitalised PHN. The primary endpoints were hospitalised HZ (primary diagnosis) and hospitalised PHN (primary diagnosis). The secondary endpoints also included hospitalised HZ (secondary diagnosis), hospitalised PHN (secondary diagnosis) and community HZ (antiviral prescription). Hospitalised HZ is a unilateral dermatomal vesicular disease associated with radicular pain requiring hospitalisation. It was identified as the International Statistical Classification of Diseases and Related Health Problems, Tenth Revision, Australian Modification (ICD-10-AM-iii) Code B02 (primary and secondary diagnoses) in the NMDS (Table S2). Compared to review of medical records, the use of ICD coded data has a positive predictive value of 85–91%.^25^^,^^26^ Hospitalised PHN is persistent debilitating pain ≥ 90 days after the acute phase of painful dermatomal vesicular rash requiring hospitalisation. It was identified using ICD-10-AM-iii, code G530 (primary and secondary diagnoses) recorded in the NMDS (Table S2). PHN diagnosis codes associated with an encounter (hospitalisation) have a positive predictive value of 73–89%.^27^ The NMDS contains the following: Event ID; Clinical code system (ICD-10-AM-iii); Clinical code; Diagnosis type; Diagnosis sequence; and Submitted system ID. First, we looked at events that list the codes for HZ and PHN, then looked at the field ‘diagnosis type’. An ‘A’ indicates that this was the primary diagnosis. A ‘B’ indicates that this was a secondary diagnosis.

Community HZ is unilateral dermatomal vesicular disease associated with radicular pain treated in the outpatient department or primary care service with antiviral drugs. It was identified using the pharmaceutical collection. The prescription of 800 mg (tab or tab dispersible; Formulation ID 248305 or 248308) of acyclovir five times daily or 1000 mg (Formulation ID 104325) of valacyclovir three times daily, for seven days prescription schedule for HZ, is hereafter referred to as a shingles prescription. This is the recommended and fully subsidised treatment of HZ in New Zealand.^28^^,^^29^

### Risk factors

Any individual with congenital or acquired immunodeficiency was considered immunosuppressed (Table S4). Cancer was defined using the morphological codes in the Cancer Registry. Chronic co-morbidities included chronic obstructive pulmonary diseases (COPD), liver disease, diabetes mellitus (DM), kidney disease, cerebrovascular disease (including stroke), ischaemic heart disease and autoimmune diseases. An individual could have one or more chronic diseases (Table S5).

### Falsification outcomes

There may be differences between vaccinated and unvaccinated cohorts in terms of measurable (but not measured) and unmeasurable confounders.^30^ These factors may range from underlying baseline characteristics to health seeking behaviour (or access to health care).^31^^,^^32^ We assessed for bias by calculating the hazard ratio of six conditions (renal calculi, cholelithiasis and cholecystitis, burns, haemorrhoids, epistaxis, and pancreatic diseases) requiring hospitalisation and not associated with HZ (Figure S3). Clustering of the measure of association around zero indicated that these cohorts were comparable.

### Statistical analysis

Vaccination status was considered a time-varying exposure because the vaccination status of individuals over time could remain the same (unvaccinated) or change from unvaccinated to vaccinated. Therefore, vaccinated individuals contributed both unvaccinated time-at-risk (before vaccination) and vaccinated time at risk for occurrence of outcomes of interest. The start of vaccinated person-time at risk (index date) was the vaccination date plus 30 days. A window period of 30–42 days is required for the vaccine to take effect in the vaccinated cohort.^33^, ^34^, ^35^

Baseline patient characteristics included age; sex; prioritised ethnicity - identified using level 1 ethnicity codes (Table S3); quintiles of the NZ Deprivation Index (one = most deprived and five = least deprived); immune status including congenital or acquired immunodeficiency (Table S4), and cancer defined using the morphological codes in the Cancer Registry; co-morbidities included COPD, liver disease, DM, kidney disease, cerebrovascular disease (including stroke), ischaemic heart disease and autoimmune diseases (seropositive and other rheumatoid arthritis, psoriatic and enteropathic arthropathies, systemic lupus erythematosus, irritable bowel syndrome, psoriasis, ankylosing spondylitis (Table S5); and District Health Board were presented as frequencies and percentages for both matched study cohorts (vaccinated and unvaccinated).

Overall and risk factor specific crude incidence rates (hereafter referred to as incidence rate), and 95% confidence intervals (CIs) of HZ and PHN for vaccinated and unvaccinated groups were estimated by dividing the number of incident cases (retrieved from the NMDS and pharmaceutical collections) by the total person-time of follow-up. The index date for the vaccinated cohort was the date of vaccination (retrieved from the NIR) plus 30 days. We made the assumption that the date recorded in the NIR is the date the person received the vaccine. Each vaccinated person was followed from the index date until the development of the outcome of interest or the end of the study (31^st^ March 2021). Unvaccinated were followed from the start of the study (1^st^ April 2018) until the development of outcome of interest or end of the study.

Cox proportional hazards model was used to derive hazard ratios (and 95% CIs) for HZ and PHN in the vaccinated compared to the unvaccinated adjusting for potential confounders. Immunosuppression and co-morbidities were treated as timefixed binary covariates. Schoenfeld residuals and plots of Martingale residuals against continuous covariates were used to assess proportional hazards and nonlinearity, respectively.

We calculated VE as one minus adjusted hazard ratio (aHR) times 100 [(1-aHR) * 100]. Stratified aHR, VE (age, sex, co-morbidity, ethnicity, NZDep2013, and immune status) were calculated, including 95% CIs. For outcomes of interest with small numbers, some subgroup analyses resulted in estimates with low levels of statistical precision, and subgroups were combined. The analyses were carried out by JM and AW and independently checked by a statistician (BN). Statistical analyses were carried out using R/R Studio (version R-4.0.5) and Python (version 3.7.12).^36^^,^^37^

### Ethics and permissions

An ethics exemption (21/NTB/118) was obtained from the Health and Disability Ethics Committee of the Ministry of Health, New Zealand.

### Role of the funding source

The sponsors of the study had no role in the study design, data collection, data analysis, data interpretation, or the writing of this report.

## Results

### Baseline characteristics

The study population consisted of 274,272 vaccinated people matched with 549,870 unvaccinated (Figure S2 and Table S6). The matched population was 93.4% immunocompetent, 52.2% female, 80.2% European (level 1 ethnic codes), and 64.5% were 65–74 years old (mean age = 71.1±5.0) (Table 1). The average duration of follow-up was 2.4 years.

**Table 1:** Baseline characteristics of matched study cohorts by herpes zoster vaccination status.

Characteristics		Total *N*= 824,142		Vaccinated (%) *N* = 274,272 (33.28%)		Unvaccinated (%) *N*= 549,870 (66.72%)	
Age	45–49	121	0.01%	40	0.02%	81	0.02%
	50–54	462	0.06%	152	0.06%	310	0.06%
	55–59	757	0.09%	253	0.09%	504	0.09%
	60–64	67,615	8.20%	21,817	7.95%	45,798	8.33%
	65–69	277,163	33.63%	92,417	33.69%	184,746	33.60%
	70–74	254,506	30.88%	84,952	30.97%	169,554	30.84%
	75–76	181,177	21.98%	60,470	22.05%	120,707	21.95%
	≥ 80	42,341	5.14%	14,171	5.17%	28,170	5.12%
Sex	Male	394,051	47.81%	131,143	47.81%	262,908	47.81%
	Female	430,011	52.19%	143,126	52.18%	286,956	52.19%
Ethnicity (Level 1 ethnic codes)	Māori (2)	47,639	5.78%	15,837	5.77%	31,802	5.78%
	Pacific Peoples (3)	27,472	3.33%	9050	3.30%	18,422	3.35%
	European (1)	660,427	80.14%	222,419	81.09%	438,008	79.66%
	Asian (4)	71,506	8.68%	23,732	8.65%	47,774	8.69%
	MELAA (5)	3716	0.45%	1199	0.44%	2517	0.46%
	Other Ethnicity (6)	1531	0.19%	506	0.18%	1025	0.19%
	Residual Categories (9)	11,851	1.44%	1529	0.56%	10,322	1.88%
NZDep2013 (Quintiles)	Quin 1	165,225	20.05%	57,936	21.12%	107,289	19.51%
	Quin 2	158,834	19.27%	54,788	19.97%	104,046	18.92%
	Quin 3	168,393	20.43%	56,872	20.73%	111,521	20.28%
	Quin 4	173,984	21.11%	57,324	20.90%	116,660	21.21%
	Quin 5	157,706	19.14%	47,352	17.26%	110,354	20.07%
Immune suppression	Yes	54,834	7.13%	16,729	6.10%	38,105	6.93%
	No	769,308	93.35%	257,543	93.90%	511,765	93.07%
COPD	Yes	34,465	4.18%	10,193	3.72%	24,272	4.41%
	No	789,677	95.82%	26,4079	96.28%	525,598	95.58%
DM	Yes	84,187	10.22%	34,231	12.48%	49,956	9.08%
	No	739,955	89.78%	240,041	87.52%	499,914	90.91%
Kidney disease	Yes	31,858	3.87%	9854	3.59%	22,004	4.00%
	No	792,284	96.13%	264,418	96.41%	527,866	96.00%
Liver disease	Yes	2979	0.36%	578	0.21%	2401	0.44%
	No	821,163	99.64%	273,694	99.80%	547,469	99.56%
IHD	Yes	84,517	10.26%	32,959	12.02%	51,558	9.38%
	No	739,625	89.74%	241,313	87.98%	498,312	90.62%
CVD	Yes	39,123	4.75%	12,810	4.67%	26,313	4.78%
	No	785,019	95.25%	261,462	95.33%	523,557	95.21%
Gout	Yes	9870	1.20%	3251	1.18%	6619	1.20%
	No	814,272	98.80%	271,021	98.81%	543,251	98.79%
AI disease	Yes	5483	0.67%	1204	0.44%	4279	0.78%
	No	818,643	99.33%	273,068	99.56%	545,575	99.22%
Cancer	Yes	127,128	15.43%	41,165	15.01%	85,960	15.63%
	No	696,998	84.57%	233,107	84.99%	463,910	84.37%
DHB	Auckland	73,517	8.92%	22,149	8.08%	51,368	9.34%
	Bay of Plenty	49,482	6.0%	17,739	6.47%	31,743	5.77%
	Canterbury	96,208	11.67%	33,224	12.11%	62,984	11.46%
	Capital & Coast	45,073	5.47%	14,327	5.22%	30,746	5.59%
	Counties Manukau	73,402	8.91%	27,406	9.99%	45,996	8.37%
	Hawke's Bay	31,891	3.87%	8801	3.21%	23,090	4.19%
	Hutt Valley	23,649	2.87%	7999	2.92%	15,650	2.84%
	Lakes	19,068	2.87%	6970	2.54%	12,098	2.20%
	MidCentral	32,612	3.96%	11,691	4.26%	20,921	3.81%
	Nelson Marlborough	33,969	4.12%	11,473	4.18%	22,496	4.09%
	Northland	38,023	4.61%	12,522	4.57%	25,501	4.64%
	South Canterbury	13,637	1.65%	4649	1.70%	8988	1.63%
	Southern	61,653	7.48%	20,010	7.29%	41,643	7.57%
	Tairawhiti	7837	0.95%	2018	0.73%	5819	1.06%
	Taranaki	22,661	2.75%	7593	2.77%	15,068	2.75%
	Waikato	68,862	8.36%	22,447	8.18%	46,415	8.44%
	Wairarapa	10,617	1.29%	4284	1.56%	6333	1.15%
	Waitemata	92,716	11.25%	30,808	11.23%	61,908	11.25%
	West Coast	7412	0.90%	2502	0.91%	4910	0.89%
	Whanganui	13,721	1.66%	5559	2.03%	8162	1.48%
	Oversees and unknown	8132	0.99%	101	0.04%	8031	1.46%

NZDep2013: Index of deprivation 13 divided into five quintiles.

DM: Diabetes mellitus.

COPD: Chronic obstructive pulmonary disease.

IHD: Ischaemic heart disease.

**MELAA: Middle Eastern / Latin American / African**

CVS: Cerebrovascular diseases including stroke.

AI disease: Autoimmune diseases (seropositive and other rheumatoid arthritis, psoriatic and enteropathic arthropathies, systemic lupus erythematosus, irritable bowel syndrome, psoriasis, ankylosing spondylitis).

DHB: District Health Board.

### Incidence rates of herpes zoster and postherpetic neuralgia

During the follow-up period, there were a total of 7819 incidence HZ cases: 718 (9.2%) cases were hospitalised with HZ (primary diagnosis = 341) and 7101 (90.8%) cases received a shingles prescription (Table S7, Figure S4). Amongst the vaccinated cohort 100 people were hospitalised with HZ (incidence rate: 0.16 per 1000 person-years), and amongst the unvaccinated, 618 (incidence rate: 0.31 per 1000 person-years) were hospitalised with HZ (Table 2). There were 7101 community HZ cases identified during the entire study period (1346 in the vaccinated cohort and 5755 in the unvaccinated cohort). The incidence rate of community HZ was 2.2 (95% CI:2.08 – 2.32) and 2.87 (95% CI: 2.79 – 2.94) per 1000 person-years in the vaccinated and unvaccinated groups, respectively (Table 3). There were 180 hospitalised PHN cases (primary diagnosis = 40) identified during the study period: 19 cases in the vaccinated (incidence rate: 0.031 per 1000 person-years), and 161 cases in the unvaccinated cohorts (incidence rate: 0.080 per 1000 person-years) (Table 4).

**Table 2:** Hospitalised herpes zoster crude incidence rate by vaccinated status in the matched population.

Characteristics		Vaccinated			Unvaccinated		
		*N* = 274,272 (33.28%)			*N*=549,870 (66.72%)		
		Cases	PY	Crude incidence rate	Cases	PY	Crude incidence rate
				(95% CI) *1000 PY			(95% CI) *1000 PY
**Overall**		**100**	**614478.5**	**0.163 (0.132–0.198)**	**618**	**2015888.3**	**0.307 (0.283–0.332)**
**Age**	45–64	≤ 5	20564	0.05 (0.001–0.26)	41	195410.2	0.21 (0.15–0.28)
	≥ 65*	99	593192.5	0.17 (0.14–0.20)	577	1816920	0.32 (0.29–0.34)
	**65–69**	23	204634.8	0.11 (0.07–0.17)	184	679988.5	0.27 (0.23–0.31)
	**70–74**	32	202532.4	0.16 (0.11–0.22)	181	609601	0.30 (0.26–0.34)
	**75–76**	30	148180.7	0.20 (0.14–0.29)	160	430077.5	0.37 (0.32–0.43)
	**≥ 80**	14	37844.6	0.37 (0.20–0.62)	52	97252.8	0.53 (0.40–0.70)
**Sex**	**Male**	56	292215.2	0.19 (0.14–0.25)	278	965421.1	0.29 (0.26–0.32)
	**Female**	44	322260.0	0.14 (0.10–0.81)	340	1050442.3	0.32 (0.29–0.36)
**Ethnicity** **(Level 1 ethnic codes)**	**Māori (2)**	7	34414.7	0.20 (0.08–0.42)	42	117641.04	0.36 (0.26–0.48)
	**Pacific peoples (3)**	4	19348.8	0.12 (0.03–0.30)	21	68300.1	0.31 (0.19–0.47)
	**European (1)**	84	504348.3	0.17 (0.13–0.21)	479	1603059.2	0.30 (0.27–0.33)
	**Others (4+5+6+9)**	5	56352.6	0.09 (0.03–0.21)	41	226997.7	0.18 (0.13–0.25)
**NZDep2013** **(Quintiles)**	**1** (**least deprived)**	22	127102.0	0.17 (0.11–0.26)	104	399757.1	0.26 (0.21–0.32)
	**2**	19	122201.5	0.16 (0.09–0.24)	111	384536.3	0.29 (0.24–0.35)
	**3**	23	128271.3	0.18 (0.11–0.27)	149	409062.4	0.36 (0.31–0.43)
	**4**	17	129935.9	0.13 (0.08–0.21)	151	425449.0	0.35 (0.30–0.42)
	**5 (most deprived)**	19	106967.9	0.18 (0.11–0.28)	101	397083.9	0.25 (0.21–0.31)
**Immune status**	**Competent**	79	575565	0.14 (0.11–0.17)	464	1879714.4	0.25 (0.22–0.27)
	**Suppressed**	21	38913.6	0.54 (0.33–0.82)	151	136174.4	1.11 (0.94–1.30)
**Comorbidities**	**COPD^b^**	13	24230.7	0.54 (0.29–0.92)	87	85797.5	1.01 (0.81–1.25)
	**Diabetes**	31	79453.3	0.39 (0.27–0.55)	132	188273.4	0.70 (0.59–0.83)
	**Kidney disease**	17	23605.7	0.72 (0.42–1.15)	93	78065.0	1.19 (0.96–1.46)
	**Liver disease**	≤ 5	1367.2	2.19 (0.45–6.41)	13	8164	1.59 (0.85–2.72)
	**IHD**	22	77111.8	0.29 (0.18–0.43)	144	191675.5	0.75 (0.63–0.88)
	**CVS**	19	30290.3	0.63 (0.38–0.98)	69	94563.7	0.73 (0.57–0.92)
	**Gout**	7	7491.4	0.93 (0.38–1.93)	27	23969.9	1.13 (0.74–1.64)
	**AI disease**	≤ 5	2801.3	0.36 (0.01–1.99)	16	14746	1.09 (0.62–1.76)
	**Cancer**	27	95709.3	0.28 90.19 – 0.41)	160	310184.5	0.52 (0.44 – 0.66)

Age group ≥ 65 years*: We have reported the overall crude incidence for 65+ given that many current observational studies focus on 65+ population.

Hospitalised zoster (primary and secondary): A publicly funded hospital discharge between 1 January 2018 and 31 March 2021 (inclusive) with any diagnosis of Zoster [herpes zoster] (ICD-10-AM-iii code B02).

NZDep2013: Index of deprivation 13 divided into five quintiles.

Others = Asian (4), **Middle Eastern / Latin American / African-** (5), Other Ethnicity (6), Residual Categories (9).

COPD: Chronic obstructive pulmonary disease,

IHD: Ischaemic heart disease.

CVS: Cerebrovascular diseases including stroke,

AI disease: Autoimmune diseases (Seropositive and other rheumatoid arthritis, psoriatic and enteropathic arthropathies, systemic lupus erythematosus, irritable bowel syndrome, psoriasis, ankylosing spondylitis).

Excluded groups: Too few observations to result in stable estimates (small numbers of cases per group preclude subgroup analysis for these variables).

PY: Person-years.

Crude incidence rate and associated confidence intervals were calculated using the epiR package.

Quintiles: Quintile 1 represents areas with the least deprived scores and Quintile 10 represents areas with the most deprived scores.

**Table 3:** Community Herpes Zoster (shingles prescription) incidence by Vaccinated Status in the Matched population.

Characteristics		Vaccinated			Unvaccinated		
		*N* = 274,272 (33.28%)			*N*=549,870 (66.72%)		
		Cases	PY	Crude incidence rate	Cases	PY	Crude incidence rate
				(95% CI) *1000 PY			(95% CI) *1000 PY
**Overall (45+ years)**		1346	612857.8	2.20 (2.08–2.32)	5755	2008898.9	2.87 (2.79–2.94)
Overall (≥ 65 years) *		1312	591593.5	2.22 (2.10–2.34)	5115	1810943	2.82 (2.75–2.90)
**Age**	**45 – 64**	34	21264.3	1.60 (1.11–2.23)	640	197955.4	3.23 (2.99–3.49)
	**65 – 69**	417	204097.0	2.04 (1.85–2.25)	1959	677490.4	2.89 (2.76–3.02)
	**70 – 74**	461	202010.1	2.28 (2.28–2.50)	1694	607677.2	2.79 (2.66–2.92)
	**75 – 79**	323	147759.2	2.19 (1.95–2.44)	1208	428699.9	2.82 (2.66–2.98)
	**≥ 80**	111	37727.2	2.94 (2.42–3.54)	254	97075.9	2.62 (2.30–2.96)
**Sex**	**Male**	552	291556.3	1.89 (1.74–2.06)	2455	962432.9	2.55 (2.45–2.65)
	**Female**	794	321301.6	2.47 (2.30–2.65)	3300	1046465.9	3.15 (3.05–3.26)
**Ethnicity (Level 1)**	**European**	1123	502993.0	2.23 (2.10–2.37)	4835	1597144.6	3.03 (2.94–3.11)
	**Māori**	75	34322.6	2.19 (1.72–2.74)	339	117231.2	2.89 (2.59–3.22)
	**Pacific Peoples**	29	19314.0	1.50 (1.01–2.16)	101	68223.8	1.48 (1.21–1.80)
	**Others**	119	56228.3	2.12 (1.75–2.53)	480	226299.2	2.12 (1.94–2.32)
**NZDep2013 (Quintiles)**	**1 (least deprived)**	272	126773.4	2.15 (1.90–2.42)	1086	398387.5	2.73 (2.57–2.89)
	**2**	253	121890.7	2.08 (1.83–2.35)	1183	382953.6	3.09 (2.92–3.27)
	**3**	311	127908.1	2.43 (2.17–2.72)	1307	407460.7	3.21 (3.04–3.39)
	**4**	262	129607.3	2.02 (1.78–2.28)	1245	423836.1	2.94 (2.78–3.11)
	**5 (Most deprived)**	248	106678.4	2.32 (2.04–2.63)	934	396261.0	2.36 (2.21–2.51)
**Immune status**	**Competent**	1208	574122.0	2.10 (1.99–2.23)	4991	1873775.4	2.66 (2.59–2.74)
	**Suppressed**	138	38735.8	3.56 (2.99–4.21)	764	135123.4	5.65 (5.26–6.07)
**Comorbidities**	**COPD**	72	24166.4	2.98 (2.33–3.75)	308	85589.8	3.60 (3.21–4.02)
	**Diabetes**	193	79224.6	2.44 (2.10–2.81)	651	187561.4	3.21 (3.21–3.75)
	**Kidney disease**	73	23517.7	3.10 (2.43–3.90)	280	77738.4	3.60 (3.19–4.05)
	**IHD**	197	76872.8	2.56 (2.22–2.95)	758	190885.9	3.97 (3.69–4.26)
	**CVS**	82	30198.4	2.72 (2.16–3.37)	328	94193.6	3.48 (3.12–3.88)
	**Gout**	14	7486.9	1.87 (1.02–3.14)	85	23907.5	3.56 (2.84–4.40)
	**AI diseases**	11	2785.1	4.31 (2.23–7.53)	65	14634.7	5.12 (4.03–6.42)
	**Cancer**	221	95458.3	2.32 (2.02–2.64)	1046	308821.6	3.39 (3.18–3.60)

Age group ≥ 65 years*: We have reported the overall crude incidence for 65+ given that many current observational studies focus on 65+ population.

Crude incidence was estimated using the epiR package; PY= person-years

Others = Asian (4), Middle Eastern/Latin American/African (5), Other Ethnicity (6), Residual Categories (9)

Community HZ= Antiviral prescription (Formulation ID: 248305; 248308 and 104325)

NZDep2013= NZ deprivation quintiles is a measure of area-based socioeconomic deprivation, was divided into quintiles (Quin 1 = least deprived; Quin 5 = most deprived)

COPD=Chronic obstructive pulmonary disease

CVS=Cerebrovascular diseases

IHD=Ischaemic heart disease

AI diseases =Autoimmune diseases.

Excluded groups: Too few observations to result in stable estimates.

**Table 4:** Hospitalised postherpetic neuralgia incidence by vaccinated status in the matched population.

Characteristics		Vaccinated			Unvaccinated		
		*N* = 274,272 (33.28%)			*N*=549,870 (66.72%)		
		Cases	PY	Crude incidence rate	Cases	PY	Crude incidence rate
				(95% CI) *1000 PY			(95% CI) *1000 PY
**Overall**		**19**	**618347.1**	**0.031 (0.019**–**0.048)**	**161**	**2025700.5**	**0.080 (0.068**–**0.093)**
**Age (years)**	**45**–**74**	8	431037.9	0.019 (0.008–0.037)	109	1496044.5	0.073 (0.060–0.088)
	**≥ 75**	11	187309.1	0.059 (0.029–0.105)	52	529656.1	0.098 (0.073–0.129)
**Sex**	**Female**	12	324440.7	0.037 (0.019–0.065)	88	1056329.5	0.083 (0.067–0.103)
	**Male**	7	293906.4	0.024 (0.010–0.049)	73	969371.0	0.075 (0.059–0.095)
**Ethnicity**	**Others**	≤ 5	56705.8	0.0353(0.004–0.127)	6	227701.0	0.026 (0.010–0.057)
	**Māori**	≤ 5	34667.3	0.029 (0.001–0.161)	8	118110.2	0.068 (0.029–0.133)
	**Pacific Peoples**	0	19439.2	0.00	≤ 5	68476.6	0.058 (0.016–0.150)
	**European**	16	507532.9	0.032 (0.018–0.051)	140	1611391.5	0.087 (0.073–0.103)
**NZDep2013 (Quintiles)**	**Quin 1**	≤ 5	127909.8	0.024 (0.005–0.069)	36	401907.6	0.090 (0.063–0.124)
	**Quin 2**	≤ 5	122888.7	0.024 (0.005–0.071)	22	386470.4	0.057 (0.036–0.086)
	**Quin 3**	≤ 5	129135.4	0.031 (0.008–0.079)	41	411359.1	0.100 (0.071–0.135)
	**Quin 4**	≤ 5	130776.3	0.031 (0.008–0.078)	32	427435.6	0.075 (0.051–0.106)
	**Quin 5**	≤ 5	107639.9	0.047 (0.015–0.108)	24	398524.7	0.060 (0.039–0.090)
**Immune status**	**Competent**	16	579113.7	0.028 (0.016–0.045)	117	1888149.2	0.062 (0.051–0.074)
	**Suppressed**	≤ 5	39233.4	0.077 (0.016–0.224)	44	137551.3	0.340 (0.232–0.429)
**Comorbidity**	**DM**	6	79984.5	0.075 (0.028–0.163)	36	189461.8	0.190 (0.133–0.263)
	**IHD**	6	77679.4	0.077 (0.028–0.168)	34	193047.4	0.176 (0.122–0.246)
	**CVS**	6	30533.3	0.196 (0.072–0.428)	20	95181.4	0.210 (0.128–0.325)
	**Cancer**	≤ 5	96350.2	0.052 (0.017–0.121)	49	312000.8	0.157 (0.116–0.208)

Hospitalised postherpetic neuralgia (primary and secondary diagnosis): A publicly funded hospital discharge between 1 January 2018 and 31 March 2021 (inclusive) with any diagnosis of postherpetic neuralgia (ICD-10-AM-iii code G530).

Others = Asian (4), **Middle Eastern / Latin American / African** (5), Other Ethnicity (6), Residual Categories (9).

Excluded groups: Too few observations to result in stable estimates (small numbers of cases per group preclude subgroup analysis for these variables).

PY: Person-years.

Crude incidence and associated confidence intervals were calculated using the epiR package.

### Herpes zoster vaccine effectiveness

#### Herpes zoster

In the adjusted analysis, HZ vaccination was associated with reduced HZ hospitalisation, community HZ and PHN hospitalisation (Table 5, Table 6). In the primary analysis, the adjusted VE for ZVL against hospitalised HZ was 57.8% (95% CI: 41.1–69.8) in adults ≥ 45 years old and 54.4% (95% CI: 36.0–67.5) in adults ≥ 65 years old.

**Table 5:** Herpes zoster vaccine effectiveness against herpes zoster by characteristics and disease definition.

Characteristic (Vaccination status)		Community HZ* (Shingles prescription)		Hospitalised HZ* (primary diagnosis)		Hospitalised HZ* (primary and secondary)	
		Adj. HR (95% CI)	VE (95% CI)	Adj HR (95% CI)	VE (95% CI)	Adj. HR (95% CI)	VE (95% CI)
**Vaccination status (45+)**	**Unvaccinated**	Reference		Reference		Reference	
	**Vaccinated**	0.700 (0.665–0.750)	30.0% (25.0–33.5)	0.422 (0.302–0.589)	57.8% (41.1–69.8)	0.514 (0.415–0.637)	48.6% (36.3–58.5)
**Vaccination status (65-80)**	**Unvaccinated**	Reference		Reference		Reference	
	**Vaccinated**	0.698 (0.655–0.744)	30.2% (25.6–34.5)	0.418 (0.293–0.572)	58.2% (42.8–70.7)	0.497 (0.394–0.627)	50.3% (37.3–60.6)
**Age group**	**45**–**64**	0.422 (0.297–0.598)	57.8% (40.2–70.3)	0.189 (0.00–inf)	81.1% (0.00–inf)	0.227 (0.031–1.679)	77.3% (−67.9 to 96.9)
	≥ 65*	0.721 (0.679–0.767)	27.8% (23.3–32.1)	0.456 (0.325–0.640)	54.4% (36.0–67.5)	0.515 (0.415–0.639)	48.5% (36.1–58.5)
	**65**–**69**	0.643 (0.578–0.715)	35.7% (28.5–42.2)	0.338 (0.170–0.674)	66.2% (32.6–83.0)	0.417 (0.271–0.650)	58.3% (35.0–72.9)
	**70 – 74**	0.752 (0.677–0.834)	24.8% (16.6–32.3)	0.388 (0.206–0.731)	61.2% (26.9–79.4)	0.528 (0.361–0.772)	47.2% (22.8–63.9)
	**75 – 79**	0.718 (0.634–0.813)	28.3% (18.7–36.6)	0.532 (0.304–0.932)	46.8% (6.8–69.6)	0.558 (0.375–0.828)	44.2% (17.2–62.5)
	**80+**	1.03 (0.822–1.29)	−3.0% (−29.0 to 17.8)	0.511 (0.193–1.35)	48.9% (−0.35 to 80.7)	0.645 (0.356–1.170)	35.5% (−17.0 to 64.4)
**Sex**	**Female**	0.723 (0.669–0.783)	27.7% (21.7–33.1)	0.389 (0.246–0.614)	61.1% (38.6–75.4)	0.411 (0.300–0.565)	58.9% (43.5–70.0)
	**Male**	0.680 (0.619–0.747)	32.0$ (25.3–38.1)	0.463 (0.284–0.756)	53.7% (24.4–71.6)	0.640 (0.478–0.857)	36.0% (14.3–52.2)
**Ethnicity** **(Level 1)**	**Māori**	0.680 (0.528–0.877)	32.0% (12.5–47.2)	0.341(0.078–1.48)	65.9% (−48 to 92.2)	0.548 (0.244–1.232)	45.2% (−23.2 to 75.6)
	**Pacific Peoples**	0.845 (0.554–1.29)	15.5% (−29.0 to 44.6)	0.702 (0.145–3.38)	29.8% (−238 to 85.5)	0.478 (0.163 –1.406)	52.2% (−40.6 to 83.7)
	**European**	0.688 (0.645–0.735)	31.2% (26.5–35.5)	0.412 (0.286–0.595	58.8% (40.5–71.4)	0.524 (0.414–0.662)	47.6% (33.8–58.6)
	**Others**	0.833 (0.678–1.02)	16.7% (−2.0 to 32.2)	0.435 (0.128–1.47)	56.5% (−47 to 87.2)	0.370 (0.182–0.752)	63.0% (24.8–81.8)
**NZDep2013** 1 = “least deprivation” 5= “most deprivation”	**Quin 1**	0.732 (0.640–0.838)	26.8% (16.2–36.0)	0.463 (0.227–0.945)	53.7% (5.5–77.3)	0.617 (0.387–0.984)	38.3% (1.6–61.3)
	**Quin 2**	0.638 (0.556–0.732)	36.2% (26.8–44.4)	0.490 (0.231–1.04)	51.0% (−4.0 to 76.9)	0.591 (0.360–0.971)	40.9% (2.9–64.0)
	**Quin 3**	0.694 (0.612–0.786)	30.0% (21.4–38.8)	0.513 (0.268–0.981)	48.7% (1.9–73.2)	0.469 (0.301–0.731)	53.1% (26.9–69.9)
	**Quin 4**	0.632 (0.552–0.724)	36.8% (27.6–44.8)	0.208 (0.084–0.518)	79.2% (48.2–91.6)	0.362 (0.218–0.601)	63.8% (39.9–78.2)
	**Quin 5**	0.880 (0.764–1.01)	12.0% (−1.0 to 23.6)	0.512 (0.229–1.14)	48.8% (−14.0 to 77.1)	0.630 (0.383–1.035)	37.0% (−3.5 to 61.7)
**Immune status**	**Competent**	0.708 (0.664–0.755)	29.2% (24.5–33.6)	0.423 (0.293–0.612)	57.7% (38.8–70.7)	0.524 (0.412–0.668)	47.6% (33.2–58.8)
	**Suppressed**	0.668 (0.561–0.794)	33.2% (24.3–46.9)	0.400 (0.200–0.882)	60.0% (11.8–80.0)	0.485 (0.305–0.769)	51.5% (23.1–69.5)
**Comorbidities**	**COPD**	0.734 (0.566–0.952)	26.6% (0.05–43.4)	0.373 (0.112–1.24)	62.7% (−24.0 to 88.8)	0.524 (0.290–0.946)	47.6% (5.4–70.0)
	**Diabetes**	0.670 (0.568–0.789)	33.0% (21.0–43.2)	0.865 (0.235–3.19)	13.0% (−219 to 76.5)	0.620 (0.415–0.926)	38.0% (7.4–58.5)
	**Gout**	0.480 (0.272–0.850)	52.0% (15.0–72.8)	0.989 (0.189–5.16)	1.1% (−416 to 81.1)	0.809 (0.347–1.885)	19.1% (−88.5 to 65.3)
	**CVS**	0.711 (0.557–0.909)	28.9% (9.1–44.3)	0.702 (0.259–1.90)	29.8% (−90 to 74.1)	0.830 (0.496–1.389)	17.0% (−38.9 to 50.4)
	**IHD**	0.608 (0.518–0.713)	39.2% (28.7–48.2)	0.337 (0.152–0.750)	66.3% (25–84.8)	0.425 (0.269–0.671)	57.5% (32.9–73.1)
	**Cancer**	0.646 (0.558–0.748)	35.4% (25.2–44.2)	0.742 (0.091–6.03)	25.8% (−503 to 91)	0.569 (0.376–0.859)	43.1% (14.1–62.4)

Vaccination status (65–80): In Aotearoa New Zealand, the single-dose zoster vaccine live (also known as the live-attenuated zoster vaccine) was approved on the 1st of April 2018 for use in adults ≥ 50 years old and is available for no charge, to people aged ≥ 65 years (with a catchup for ages 66–80 years which ran from 2018 to 2021). It is therefore important to perform a subgroup analysis in the funded group.

Age group ≥ 65 years*: We have reported the overall adjusted VE estimate for 65+ given that many current observational studies focus on 65+ population. In the subgroup analysis of hospitalised herpes zoster (primary diagnosis), the age group 45–64 years had too few observations to result in stable estimates.

Community HZ= Antiviral prescription (Formulation ID: 248305; 248308 and 104325).

Primary diagnosis = Condition chiefly responsible for the patient's admission to the hospital (first-listed diagnosis).

Secondary diagnosis= Coexist at the time of admission, that develop subsequently, or that affect the treatment received and/or length of stay.

NZDep2013= NZ deprivation quintiles is a measure of area-based socioeconomic deprivation, was divided into quintiles (Quin 1 = least deprived; Quin 5 = most deprived).

COPD=Chronic obstructive pulmonary disease.

CVS=Cerebrovascular diseases; IHD=Ischaemic heart disease.

In the subgroup analysis, most groups had too few observations to result in stable estimates (small numbers of cases per group preclude subgroup analysis for these variables).

Hazard ratio was adjusted for age, sex, ethnicity, index of deprivation, immune status, and comorbidities.

**Table 6:** Herpes zoster vaccine effectiveness against postherpetic neuralgia by characteristics and disease definition.

Primary diagnosis		Adjusted HR		VE (95% CI)
Overall (≥45 years)		0.263 (0.080–0.860)		73.7% (14.0–92.0)
Overall (65–80 years)		0.258 (0.079–0.848)		74.2% (15.2–92.1)
Overall (≥ 65 years)*		0.245 (0.075–0.801)		75.5% (19.9% – 92.5)
**Primary and secondary**				
Overall (≥ 45 years)		0.372 (0.230–0.600)		62.8% (40.0–77.0)
Overall (65–80 years)		0.341 (0.203–0.573)		65.9% (42.7–79.7)

Primary and secondary diagnoses				
Characteristics		Adjusted HR (95% CI)33	VE (95% CI)	
**Age (years)**	**45**–**64**	2.6×10 ^−8^ (0.00–Inf)	Value cannot be reliably calculated	
	≥ 65*	0.371 (0.230–0.600)	62.9% (40.0–77.0)	
**Sex**	**Male**	0.293 (0.134–0.638)	70.7% (36.2–86.6)	
	**Female**	0.437 (0.238–0.803)	56.3% (19.7–76.2)	
**Ethnicity**	**Māori**	0.413 (0.0512–3.340)	56.3 % (−234 to 94.9)	
	**Pacific Peoples**	1.69×10^−6^ (0.00–Inf)	Value cannot be reliably calculated	
	**European**	0.366 (0.217–0.616)	63.4% (38.4–78.3)	
	**Others**	0.573 (0.1177–2.788)	42.7% (−178.8 to 88.2)	
**NZDep2013 (Quintiles)**	**Quin 1**	0.240 (0.074–0.780)	79.0% (22.0–92.6)	
	**Quin 2**	0.499 (0.147–1.701)	50.1% (−70.1 to 85.3)	
	**Quin 3**	0.295 (0.105–0.828)	70.5% (17.2–89.5)	
	**Quin 4**	0.361 (0.127–1.028)	63.9% (−2.8 to 87.3)	
	**Quin 5**	0. 606 (0.231–1.589)	39.4% (−58.9 to 76.9)	
**Immune status**	**Competent**	0.386 (0.225–0.663)	61.4% (33.7–77.5)	
	**Suppressed**	0.324 (0.116–0.907)	67.6% (09.3–88.4)	
**Comorbidity**	**Diabetes**	0.393 (0.164–0.941)	60.7% (05.9–83.6)	
	**Cancer**	0.348 (0.138–0.879)	65.2% (12.1–86.2)	

Overall (65–80 years): In Aotearoa New Zealand, the single-dose zoster vaccine live (also known as the live-attenuated zoster vaccine) was approved on the 1st of April 2018 for use in adults ≥ 50 years old and is available for no charge, to people aged ≥ 65 years (with a catchup for ages 66–80 years which ran from 2018 to 2021). It is therefore important to perform a subgroup analysis in the funded group.

Overall (≥ 65 years)*: We have reported the overall adjusted VE estimate for 65+ given that many current observational studies focus on 65+ population. In the subgroup analysis, the age group 45–64 years had too few observations to result in stable estimates (small numbers of cases per group preclude subgroup analysis for these variables).

Primary diagnosis = Condition chiefly responsible for the patient's admission to the hospital (first-listed diagnosis).

Secondary diagnosis= Coexist at the time of admission, that develop subsequently, or that affect the treatment received and/or length of stay.

NZDep2013= NZ deprivation quintiles is a measure of area-based socioeconomic deprivation, was divided into quintiles (Quin 1 = least deprived; Quin 5 = most deprived).

Suppressed = mild immunosuppression.

Others = Asian (4), **Middle Eastern / Latin American / African** (5), Other Ethnicity (6), Residual Categories (9).

In the secondary analysis, the VE against hospitalised HZ was 48.6% (95% CI: 36.3–58.5) in adults ≥ 45 years. The VE against hospitalised HZ was 77.3% (95% CI: -67.9–96.9) and 48.5% (95% CI: 36.1–58.5) in adults 45–64 years and ≥ 65 years old respectively. The VE was lowest in the age group ≥ 80 years (VE = 35.5%; 95% CI: -17.0–64.4).

Amongst people with diabetes, ZVL VE against hospitalised HZ was 38.0% (95% CI: 7.4–58.5). VE was 47.6% (95% CI: 5.4–70.0) for people with COPD and 57.5% (95% CI: 32.9–73.1) for those with ischaemic heart disease. For those who had immunocompromising conditions VE was 51.5% (95% CI: 23.1–69.5).

#### Postherpetic neuralgia

In the primary analysis, adjusted VE against hospitalised PHN was 73.7 (95% CI: 14.0–92.0) in adults ≥ 45 years old and 75.5% (95% CI: 19.9–92.5) in adults ≥ 65 years old. Subgroup analyses were not done in the primary analysis. In the secondary analysis the VE against PHN was 62.8% (95% CI: 40.0–77.0) (≥ 45 years old) and 62.9% (95 CI: 40.0–77.0) (≥ 65 years old). In the subgroup analyses, ZVL was effective in preventing PHN hospitalisation in people with diabetes (VE = 60.7%; 95% CI: 5.9–83.6) and immunocompromising conditions (VE= 67.6%; 95% CI: 9.3–88.4).

#### Community herpes zoster

For community HZ, the adjusted VE was 30.0% (95% CI: 25.0–33.5) in adults ≥ 45 years. In the stratified analysis, the VE against community HZ in adults 45–64 years old was 56.8% (95% CI: 38.4 –69.7) and 27.8% (95% CI: 23.3–32.1) in adults ≥ 65 years old. In adults aged 80 years above, the VE for ZVL against community HZ was −5.1% (95% CI: -34.7–18.0) (Table 5). The ZVL was effective in preventing community HZ in people with comorbidities: immunocompromising conditions (VE = 33.2%; 95% CI: 24.3–46.9); diabetes (VE = 33.0%; 95% CI: 21.0–43.2); COPD (VE = 26.6%; 95% CI: 0.05–43.4); cerebrovascular diseases including stroke (VE = 28.9%; 95% CI: 9.1–44.3); and ischaemic heart disease (VE =39.2%; 95% CI: 28.7–48.2).

#### Māori and Pacific peoples

The study included 47,639 (5.8%) Māori and 27,472 (3.3%) Pacific peoples (Table 1). Among Māori, the incidence was lower in the vaccinated population (incidence rate: 0.20 per 1000 person- years) when compared to the unvaccinated population (incidence rate: 0.36 per 1000 person-years). The secondary analysis found a VE for community HZ of 32.0% (95% CI: 16.2 – 47.2) and VE for HZ hospitalisation was 45.3 (95% CI: -23.2–75.6). The VE against hospitalised PHN was 56.3 % (-234·0 – 94.9)

Amongst the vaccinated Pacific peoples, less than or equal to five people were hospitalised with HZ (incidence rate: 0.12 per 1000 person-years), and amongst the unvaccinated, 21 were hospitalised with HZ (incidence rate: 0.31 per 1000 person-years). The VE for ZVL against HZ hospitalisation was 52.2% (95% CI: -40.6–83·7%) and against community HZ was 15.5 (95% CI: -29.0 – 44.6) (Table 5).

## Discussion

This large nationwide retrospective matched cohort study found that zoster vaccine live vaccine effectiveness for herpes zoster hospitalisation was 57.8%, and hospitalised postherpetic neuralgia was 73·7% in New Zealand adults. The vaccine effectiveness against community herpes zoster was 30.0%. Amongst immunocompromised adults, the zoster vaccine live was effective in reducing the risk of herpes zoster hospitalisation (51.1%), postherpetic neuralgia hospitalisation (65.2%) and community herpes zoster (33.2%).

Our VE estimates were similar to those reported in randomised clinical trials (RCT). Many previous RCTs did not distinguish between community-treated herpes zoster (outpatient) and herpes zoster hospitalisation. In a meta-analysis involving five RCTs and 62,529 adults ≥ 50 years, the vaccine efficacy for ZVL against suspected herpes zoster was 39% (95% CI: 7–52). In the same study, a network meta-analysis of two RCTs (including 61,294 patients) found a vaccine efficacy of 57% (95% CI: -61–84) against confirmed (laboratory or doctor) herpes zoster. The vaccine efficacy against PHN was 66% (95% CI: 49–79).^14^ In another network meta-analysis by McGirr et al, the vaccine efficacy for ZVL against herpes zoster was 51% (95% CI: 34–47) in adults ≥ 60 years old and 37% in those ≥ 70 years and older. Also, the vaccine efficacy against PHN was 66% and 67% in adults ≥ 60 years and ≥ 70 years respectively.^10^ In the 2019 updated Cochrane review (24 studies) involving 88,531 adults 60 years and older, the vaccine efficacy against herpes zoster for ZVL was 51% (95% CI: 44–57).^38^ Variation in VE estimates may stem from variation in case definition, surveillance, and identification of active cases (mild, and severe cases), vaccine characteristics, unmeasured confounding, health system characteristics, and heterogeneity of our study population.^39^, ^40^, ^41^ Generally, the inclusion criteria for RCTs are relatively strict with exclusion of frail, immunocompromised, people with comorbidities, and/or a history of herpes zoster who would be targeted for vaccination.^16^

The effectiveness of ZVL has been assessed in several observational studies, mostly in North America, and Europe. VE estimates were largely consistent with our findings. For instance, in a recent robust matched (1:1) retrospective cohort study involving 1,891,986 adults ≥ 65 years from 2007 to 2014, Izurieta et al., found that ZVL offered protection against HZ and associated complications. During the first three years post-vaccination, VE for ZVL was 33.0% and 74.0% against community HZ (outpatient herpes zoster) and hospitalised HZ, respectively. The VE against hospitalised PHN was 57.0%.^32^ The VE was adjusted for demographic and socio-economic factors, healthcare utilisation, frailty, and immunocompromising conditions.^32^ The discrepancy in VE for HZ between community and hospitalised HZ (33% vs 74%) was similar to that in our study (30.0% vs 57.8%).^32^ This discrepency may be explained by HZ in the community being less severe, and ZVL being less effective against incident mild HZ. Also, the clinical diagnosis of HZ in the community is less specific than in the hospital. Furthermore, we used antivirals as a proxy for community HZ, which is less specific than the review of medical records. People with mild HZ may also use other treatment forms, of which some may not be captured during routine clinical practice.

In Australia, a retrospective cohort study involving 82,010 adults 70–79 years old from 2017 to 2018 showed that ZVL is effective against HZ and the protection waned over time (63.5% in year 1 to 48.2% in year 2).^42^ A Cox proportional hazards model was used to calculate the VE, adjusting for age, sex, co-morbidities, sex, general practitioner visits, location, and index of deprivation. In a recent meta-analysis involving seven cohort studies (2,473,048 participants), the VE for ZVL against HZ was 45.9% (95% CI: 42.2–49.4) in adults 50 years and older. The VE against PHN was 59.7% (95% CI: 58.4 – 89.7; three cohort studies involving 4,113,125 participants).^16^

Amongst people with immunocompromising conditions, ZVL is effective in preventing HZ and associated complications. Our VE is consistent with a recent meta-analysis of two cohort studies involving more than 200,000 immunocompromised patients (VE=45%; 95% CI: 31–56). This is clinically significant because immunosuppression is a major risk factor for VZV reactivation.^3^^,^^4^ Patients with malignancy can receive ZVL 30 days before chemotherapy.^43^ Previous studies have found that people with comorbidities (diabetes, COPD, cardiovascular diseases, renal disease, and autoimmune diseases) are at increased risk of developing HZ and associated complications.^4^ We found evidence that the ZVL prevents HZ in patients with COPD (47.6%), and DM (38.0%) These VE results are consistent with those reported in a recent meta-analysis involving four studies.^16^

There was variation in VE across ethnic groups (European, Māori, and Pacific populations). A previous study in California found VE against HZ was higher among the Hispanic population (57.0%); compared with other ethnicities: Whites (48.1%); Asian/Pacific Islanders (49.7%) and African Americans (50.5%).^44^ The ethnic specific results are likely to be impacted by ethnic differences in primary care use, hospitalisation, and prevalence of relevant comorbidities. Primary care utilisation is lower in the Māori and Pacific populations compared with the rest of the population with much higher rates of reporting financial barriers to health care.^45^ Furthermore, there are significantly fewer Māori and Pacific consultations about HZ compared with the rest of the population.^6^ The reporting of financial barriers to getting prescriptions filled is more than twice as common in Māori and Pacific populations compared with other New Zealanders with this inequity also being identified in older Māori.^45^ These lower rates of primary care use and prescription fulfilment will result in an under-reporting of Māori and Pacific cases of HZ. Conversely Māori and Pacific populations have a higher hospitalisation rate then the rest of the population.^46^ Māori and Pacific populations also have higher rates of chronic conditions that could impact on VE.^47^ The combination of these possible sources of bias could result in VE under-estimation or over-estimation against community treated HZ in Māori and Pacific populations. The level of precision in our Māori and Pacific results highlight the need for this to be a specific focus of future research, involving more years of data and possibly the use of larger number of matches for Māori and Pacific records.

This is the first population-based study in Aotearoa New Zealand to assess the effectiveness of the ZVL against HZ and PHN since the start of the programme on 1^st^ April 2018 for use in adults 50 years and older. In addition, this study also had a specific focus on the effectiveness of ZVL against HZ and associated complications within the Māori and Pacific populations. We linked routinely available national datasets from the Ministry of Health and therefore we had sufficiently large sample size to provide statistical power to generate estimates of VE with good precision. Our primary outcomes (hospitalised HZ and hospitalised PHN) were defined using ICD-10-AM-iii codes routinely used for publicly funded hospital discharges. Compared to the review of hospital records, ICD codes were found to have a positive predictive value of ≥ 83%.^26^ The accuracy of diagnostic codes in identifying HZ is similar in the vaccinated and unvaccinated cohorts.^48^ We had access to fully funded community dispensed antiviral prescriptions, which could be used as a proxy to assess VE for treated HZ in the community and are likely to include both mild and severe HZ as opposed to only severe hospitalised HZ.

In New Zealand, reporting adult vaccinations is not required by law, but it is recommended, especially for vaccinations administered to age groups eligible for funded HZ vaccinations.^19^ The ZVL is administered and recorded in the GP records. There is a direct feed into the database. There is unlikely to be many misclassifications. Almost all non-elective care is through the public system, and it is free. Almost all individuals resident in New Zealand are registered with primary care and ZVL is provided free to all who are eligible.^49^ Access to hospital services is free at the point of contact. Access to secondary care is usually obtained through a general practitioner based within a primary care practice or through emergency department.

We were able to control for confounding using our study design (restriction and matching) and data analysis methods (stratification and multivariate analysis). Each vaccinated person was matched without replacement with two unvaccinated people based on age, sex, prioritised ethnicity, and NZ deprivation index. These potential risk factors for HZ and associated complications were distributed similarly between the vaccinated and unvaccinated groups. We included age, sex, ethnicity, index of deprivation, immune status, and co-morbidities in our multivariate analysis, but acknowledge that our observational study design may be susceptible to residual confounding.^39^^,^^50^

In our study, the maximum follow-up period (3.2 years) was relatively short, and therefore, the duration of protection was not assessed. This is important because clinical trials and observational studies have shown that the protection against HZ and associated complications wanes over time and offers no protection by the seventh-year post vaccination.^32^^,^^51^, ^52^, ^53^

The use of an observational study design using routinely collected clinical data has several inherent limitations.^30^ Review of medical records for confirmation outcomes (HZ, PHN) and comorbidities was infeasible. We had no control over the data collection methods, and therefore, we could not assess the impact of several confounding factors (like education, smoking, body mass index and severity of comorbidities) on VE as these variables were not within the available datasets. These factors may be differentially distributed between the vaccinated and unvaccinated cohorts leading to a VE under-estimation or over-estimation.

We did not match the vaccinated with the unvaccinated cohort using functional status, cognitive status, or frailty (which could prevent, patients from seeking care for HZ). A subgroup analysis for people in Nursing Homes/hospice/psychiatric facilities was not done because the information was not available in the existing datasets. Frailty may also affect the chance of being vaccinated and the vaccine related immune response.^39^ People with disability face attitudinal, physical, communication, and financial barriers and have limited access to healthcare, which may result in underestimation of vaccine effectiveness.^54^

Some risk factors for HZ and associated complications (family history of HZ, personal history of HZ) may be differentially distributed between the vaccinated and unvaccinated cohorts. There may have been misclassification between the incident and prevalent HZ. These factors were not considered in the design or analysis stages of the study. People who have had HZ in the past (or prior knowledge) are more likely to receive the vaccine leading to an accurate estimate of VE.^39^ Health practitioners are more likely to talk about and recommend ZVL to people at risk of developing HZ and associated complications.^30^^,^^39^ The effect of these confounders may be minimal because both cohorts are comparable (clustering of HR around one in the falsification outcomes analysis).

In our analysis, the vaccinated cohort could not be assigned the same index date (vaccination date plus 30 days) as their matched unvaccinated cohort because the matched pairs were not flagged during data extraction. This may have led to over-estimation or under-estimation of VE. Also, access to healthcare and healthcare-seeking behaviour may be different between the vaccinated and unvaccinated cohorts and was not considered in the design or analysis of this study. Previous studies used hospital, emergency room, and physician office visits as a proxy for health-seeking behaviour. They found that vaccinated people may be healthier than unvaccinated people, leading to an inaccurate estimate of the VE.^32^^,^^39^ Although 90% of adults ≥ 55 years old with HZ seek medical care, only 22.5–36.4% (and this varies between the vaccinated and unvaccinated cohorts) of people who report persistent HZ-related pain seek medical care; hence PHN cases may be underreported.^55^^,^^56^

We used the records of fulfilment of an antiviral prescription in the pharmaceutical collection as a proxy for community-based HZ related consultations. Acyclovir and valaciclovir interfere with replication of varicella zoster virus and are fully funded (without restrictions) for management of HZ.^29^ Treatment indication was not included with these prescriptions – therefore it is possible that this prescription was for non-HZ indications. However, a small study found that 82% of HZ patients diagnosed in all NZ healthcare settings received acyclovir (the only antiviral drug prescribed for HZ in NZ during the time of this study).^28^ This outcome did not include mild cases which did not warrant treatment. We were unable to estimate the VE for community PHN as many drugs (and varied prescriptions) are used for management of PHN as well as other diseases.

General practice records are not available in the Ministry of Health database and therefore were not included in our analysis. Misclassification of cases, coding errors, and timing errors (time of care and not start of disease), misdiagnosis and vaccination status may have led to an inaccurate estimate of VE. Information about health care utilisation (GP consultations, emergency visits, specialist consultations) prior to the index data was not available, and its impact on VE was not assessed. This has been shown to influence VE in previous observational studies.^32^^,^^57^

We found lower ZVL VE for immunocompetent vs immunocompromised adults for all studied outcomes. This may be due to biases related to the selection of study participants, measurement error and residual confounding.^39^ Immunocompromised people may have fewer differential barriers to health care and are more likely to be followed closely in the health system irrespective of vaccine status. People with immunocompromising conditions may be more aware of their increased risk of developing HZ and more like to receive the ZVL. The difference is most evident in the Community HZ outcome. Vaccinated immunocompetent adults are generally motivated and therefore have more ability or desire to access care for mild HZ than the unvaccinated. Therefore, VE, especially for community HZ, is potentially more likely to be underestimated. Clinical presentation of HZ in immunocompromised people is atypical (disseminated HZ) and more likely to be misdiagnosed in the absence of a polymerase chain reaction test. Although we found evidence that the ZVL is effective against HZ and PHN in immunocompromised adults, we were unable to classify the level of immunosuppression (mild, moderate, and severe). This is important because ZVL is contraindicated in people with severe immunocompromising conditions. Also, “immunocompromised status at risk” was considered a time-fixed variable and did not change during the follow-up period. We were unable to disentangle immunocompromised status due to diseases and therapy. In a large population-based cohort study of 1.4 million adults ≥50 years, Baxter and colleagues treated immunosuppression as a time-varying covariate and found a variation in VE between the low and high immunocompromised patients.^52^

We provide the first evidence of ZVL VE against HZ and PHN in adults after the introduction of the HZ vaccination programme in Aotearoa New Zealand. Given the negative impact of HZ and associated complications on the overall quality of life, further evaluation should take place as the HZ vaccination programme matures. As other HZ vaccines become available in NZ, given the differences between real-world effectiveness and clinical trials, further studies will be needed.^14^ Future observational studies using well powered national data will also be needed to assess whether ZVL VE wanes against HZ and associated complications amongst older people in NZ. Also, there a need to understand how VE differs amongst immunosuppressed people at different stages of treatment.

## Contributors

JFM, BPN, JP and CRS conceived the idea of the study and developed the protocol. All authors reviewed the protocol. JFM, AW, and BPN cleaned and analysed the data. JFM, BPN, RP, AAS, and CRS interpreted the data. JFM wrote the first draft of the manuscript with input from AW, JP, BPN, PMAA, RP, AAS and CRS. All authors read and commented critically on drafts of the manuscript. All authors approved the final version and had final responsibility for the decision to submit for publication. CRS supervised the entire work and is the guarantor.

## Data sharing statement

The data used in this study are sensitive and will not be made publicly available.

## Declaration of interests

Professor Simpson reports grants from the UK National Institute for Health Research, UK Medical Research Council, the NZ Health Research Council and Ministry of Business Innovation and Employment. Dr. Nguyen reports grants from the NZ Ministry of Business Innovation and Employment. Dr Janine Paynter reports grants (2021) and consulting fees (2019) from GlaxoSmithKline (all grants and consulting fees were institutional), the NZ Health Research Council, the NZ Ministry of Health, and a travel grant from GAVI, The Vaccine Alliance. All other authors declare no competing interests.

## Appendix Supplementary materials

Image, application 1
