# Supplementary material for: Herpes zoster vaccine effectiveness against herpes zoster and postherpetic neuralgia in New Zealand: a retrospective cohort study
Source: Lancet Reg Health West Pac. 2022 Sep 26;31:100601. doi: 10.1016/j.lanwpc.2022.100601 (PMC9985042; doi:10.1016/j.lanwpc.2022.100601)
Supplement: Supplementary file 1 [file mmc1.docx]

**Table S1: Definition of datasets**

|  | **Datasets** | **Description** |
| --- | --- | --- |
| Demographic information | National Health Index (NHI) | Contains information needed to identify healthcare users, such as name, address (including domicile code), date of birth, sex, and ethnicity [1, 2].  Provides a mechanism for uniquely identifying every healthcare user by assigning each a unique number known as the NHI number. |
| Zoster vaccination | National Immunisation Register (NIR) | Contains data derived from unit record immunisation event information [2].  The NIR collection provides data for monitoring immunisation coverage and the progress of immunisation campaigns such as zoster. |
| Hospitalised HZ and PHN,  Diseases of interest | National Minimum Dataset (NMDS) | The NMDS is a national collection of public and private hospital discharge information, including clinical information, for inpatients and day patients [3].  Unit record data is collected and stored. All records have a valid NHI number.  It was introduced in 1999 and used for developing policy, monitoring and evaluation of performance, and scientific research. |
| Community HZ | Pharmaceutical Collection | It is a data warehouse that supports the management of pharmaceutical subsidies [4].  It contains claim and payment information from pharmacists for subsidised dispensings that have been processed by the Sector Operations General Transaction Processing System.  The pharmaceutical collection holds claims for more than 840 million scripts. |
| Mortality | Mortality Collection | Data classifying the underlying cause of death for all deaths registered in New Zealand, including all registered foetal deaths (stillbirths), using the World Health Organization Rules and Guidelines for Mortality Coding [5] .  The mortality collection provides individual level data on causes of death for research, policy development, public health surveillance, evaluation of impact public health programmes, and survival analysis. |
| Malignancies | Cancer registrations | Contains information on malignant cancer registrations which can be used for cancer incidence and survival studies, public health research, monitoring screening programmes and policy formulation [6].  Sources of data: Laboratories, Mortality collection, NMDS, and NHI |
| Socio-economic status | NZ deprivation index 2013  (NZDep2013) | Socioeconomic deprivation data and statistics [7]. It is designed to measure relative socioeconomic deprivation, not absolute socioeconomic deprivation.  Purposes: resource allocation, research, and advocacy  It combines data related to   1. Communication: Adults aged <65 without internet access at home 2. Income: Individuals aged 18-64 receiving a means tested benefit; People living in equivalised* households with income below an income threshold 3. Employment: Individuals aged 18-64 unemployed 4. Qualifications: People aged 18-64 without any qualifications 5. Owned home: People not living in own home 6. Support: People aged <65 living in a single parent family 7. Living space: People living in equivalised* households below a bedroom occupancy threshold 8. Transport: People with no access to a car   NZDep2013 provides a deprivation score for each meshblock (smallest geographical area defined by Statistics New Zealand, with a population of around 60–110 people) in New Zealand.  It is scaled from decile 1 to 10 (Quintile one to five): One = areas that have the least deprived NZDep scores; 10 = areas that have the most deprived NZDep scores. |

1. Ministry of Health. National Health Index 2020 [

2. Ministry of Health. National Immunisation Register 2021 [

3. Min J, Zhao Y, Slivka L, Wang Y. Double burden of diseases worldwide: coexistence of undernutrition and overnutrition-related non-communicable chronic diseases. Obesity Reviews. 2018;19(1):49-61.

4. Ministry of Health. Pharmaceutical Collection 2021 [Available from: <https://www.health.govt.nz/nz-health-statistics/national-collections-and-surveys/collections/pharmaceutical-collection>.

5. Ministry of Health. Mortality Collection Wellington, New Zealand: Ministry of Health; 2021 [

6. Ministry of Health. New Zealand Cancer Registry - what is collected 2021 [

7. Atkinson J, Salmond C, Crampton P. NZDep2013 index of deprivation. Wellington: Department of Public Health, University of Otago. 2014.

**
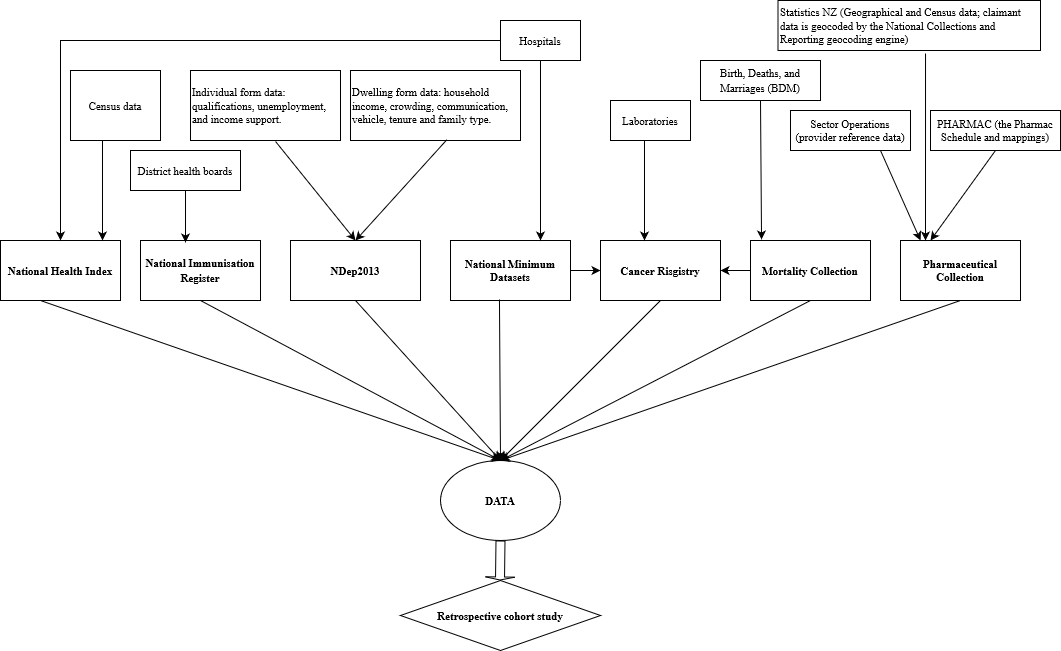
**

**Figure S1: Sources of Data**


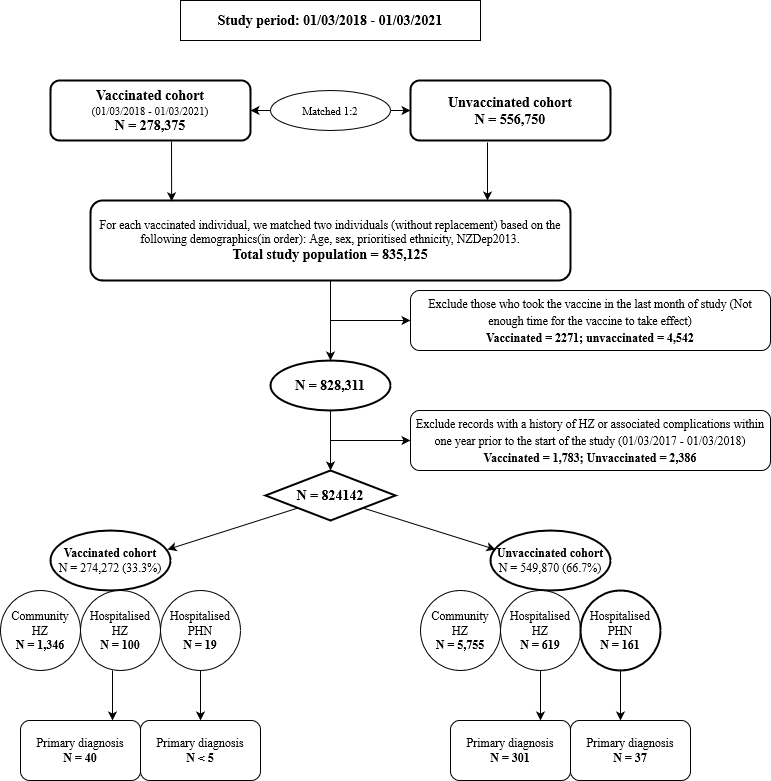


**Figure S2: Flow chart of analysis cohort**


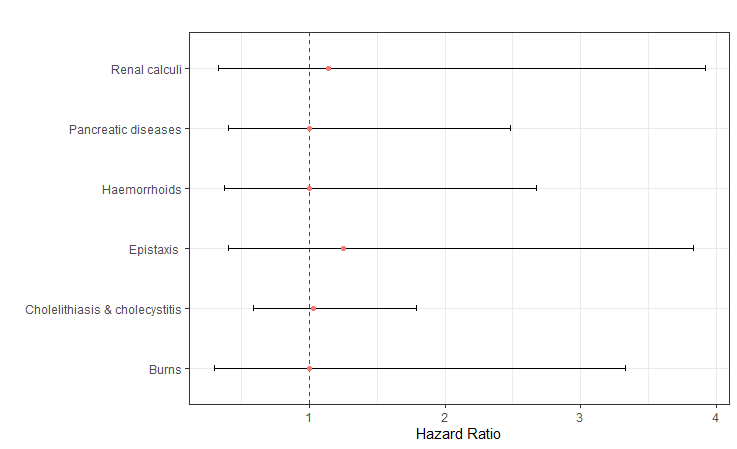


**Figure S3: Comparison of hazard ratios of six indicators conditions in the study population by ZVL vaccination status**

**Table S2: Definition of outcomes**

| **Outcome(s)** | **ICD-10-AM-iii Code** | **Code Description** |
| --- | --- | --- |
| ^*^Herpes zoster (HZ) or  Zoster  or  Shingles | B020 | Zoster encephalitis |
|  | B021 | Zoster meningitis |
|  | B022 | Zoster with other nervous system involvement |
|  | B023 | Zoster ocular disease |
|  | B027 | Disseminated zoster |
|  | B028 | Zoster with other complications |
|  | B029 | Zoster without complication |
| Herpes zoster | Formulation ID  248305 or 248308 | All government subsidised community dispensed pharmaceuticals of Aciclovir Tab 800mg or Aciclovir Tab dispersible 800mg |
|  | Formulation ID  104325 | All government subsidised community dispensed pharmaceuticals of Valaciclovir 1000mg |
| Postherpetic neuralgia (PHN) | G530 | Postzoster neuralgia |

**Table S3:** **Level 1 ethnic codes**

| **Ethnic Group code** | **Ethnic Group code description** |
| --- | --- |
| 1 | European |
| 2 | Māori |
| 3 | Pacific Peoples |
| 4 | Asian |
| 5 | Middle Eastern/Latin American/African |
| 6 | Other Ethnicity |
| 9 | Residual Categories |

**Table S4: Immune-compromising conditions**

| *Immunocompromised status as an immune response that has been weakened by disease or immunosuppressive therapy.* | |
| --- | --- |
| **Immune-compromising Condition** | **ICD-10 Code** |
| ***Congenital (primary) immunodeficiency*** | |
| Cellular immune deficiencies: *T cell, natural killer T cell, mixed cellular and antibody defects, severe combined immune deficiency (SCID)* | D80: Immunodeficiency with predominantly antibody defects  D81: Combined Immunodeficiencies  D82: Immunodeficiency associated with other major defects.  D83: Common variable immunodeficiency  D84: Other immunodeficiencies |
| ***Acquired (secondary) immunodeficiency*** | |
| Malignant Hematologic Disorders: *Blood dyscrasia, leukemia, lymphoma, other malignant neoplasms affecting bone marrow or lymphatic systems* | C81: Hodgkin lymphoma  C82: Follicular lymphoma  C83: Non-follicular lymphoma  C84: Mature T/NK-cell lymphomas  C85: Other and unspecified types of non-Hodgkin lymphoma  C86: Other specified types of T/NK-cell lymphomas  C90: Multiple myeloma and malignant plasma cell neoplasms  C91: Lymphoid leukaemia  C92: Myeloid leukaemia  C93: Monocytic leukaemia  C94: Other leukaemias of specified cell type  C95: Leukaemia of unspecified cell type  C96: Other and unspecified malignant neoplasms of lymphoid, hematopoietic, and related tissue  D75.9: Disease of blood and blood-forming organs, unspecified |
| Human Immunodeficiency Virus (HIV) Infection | B20: HIV disease resulting in infectious and parasitic disease  B21: HIV disease resulting in malignant neoplasms  B22: HIV disease resulting in other specific diseases  B23: HIV disease resulting in other conditions  B24: Unspecified HIV disease |
| Post-Solid Organ Transplantation | Z94: Transplanted organ and tissue status |
| Post-Hematopoietic Stem Cell Transplantation | Z94.8: Other transplanted organ and tissue status |
| **Immunosuppressive drugs** (Name, Formulation ID)  Abiraterone acetate (404825)  Adalimumab (383625; 383626; 383627; 383628)  Aflibercept (409825)  Alectinib (412125)  Aminoglutethimide (105501)  Amsacrine (389725; 389726)  Anagrelide hydrochloride (384325)  Anastrozole (115801)  Anastrozole-DP (388625)  Antithymocyte globulin (equine) (384125)  Antithymocyte Gobulin (375125; 375126)  Arsenic trioxide (384425; 384426; 384427)  Azacitidine (403425; 403426)  Azathioprine (110001; 110002; 110003; 110025)  Bacillus Calmette-Guerin (BCG) vaccine (392225; 392226)  Bendamustine hydrochloride (408425; 408426; 408427)  Bicalutamide (388325)  Bleomycin sulphate (381225; 381226)  Bortezomib (393625; 393626; 393627)  Busulfan (117301)  Calcium folinate (119801; 119802; 119803; 119804; 119825; 119826; 119827; 119828; 119829; 119830; 119831; 119832  Capecitabine (380825; 380826)  Carboplatin (382525; 382526; 382527; 382528; 382529)  Carmustine (383725; 383726)  Cetuximab (409425; 409426; 409427)  Chlorambucil (125501; 125502)  Ciclosporin (242101; 242102; 242103; 242125)  Cisplatin (382625; 382626; 382627)  Cladribine (384025; 384026; 384027  Colaspase [L-asparaginase] (383325; 383326)  Cyclophosphamide (136901; 136902; 136903; 136904; 136925; 136926)  Cytarabine (137101; 137102; 137103; 137104; 137125; 137126; 137127)  Dacarbazine (382725; 382726)  Dactinomycin [Actinomycin D] (384625; 384626)  Dasatinib (389425; 389426; 389427; 389428)  Daunorubicin (382825; 382826; 382827)  Docetaxel (383425; 383426; 383427; 383428; 383429; 383430; 383431)  Doxorubicin hydrochloride (381325; 381326; 381327; 381328; 381329; 381330)  Epirubicin hydrochloride (381425; 381426; 381427; 381428; 381429)  Erlotinib (391625; 391626)  Etanercept (378625; 378626; 378627)  Etoposide (243301; 243302; 243303; 243325)  Etoposide phosphate (384725; 384726)  Everolimus (404025; 404026)  Exemestane (387225)  Fludarabine phosphate (381025; 381026; 381027)  Fluorouracil (152902, 152903, 152904, 152925, 152926, 152927, 152928, 152929; 152930)  Flutamide (153701)  Fulvestrant (412625)  Gefitinib (396625)  Gemcitabine hydrochloride (384225; 384226; 384227; 384228)  Hydroxyurea [hydroxycarbamide] (162601)  Idarubicin hydrochloride (384825; 384826; 384827; 384828; 384829)  Ifosfamide (381925; 381926; 381927)  Imatinib mesylate (378025; 378026; 378027)  Infliximab (411425; 411426)  Interferon Alpha-2B (244505)  Interferon Gamma-1b (101801)  Irinotecan hydrochloride (381125; 381126; 381127)  Lapatinib ditosylate (394425)  Lenalidomide (403525; 403526; 403527; 403528)  Letrozole (118101)  Lomustine (383825; 383826)  Megestrol acetate (177201)  Melphalan (177301; 177302; 177325)  Mepolizumab (412725)  Mercaptopurine (178101; 178102; 178125)  Mesna (382025; 382026; 382027; 382028; 382029)  Methotrexate (179701; 179702; 179704; 179705; 179707; 179709; 179710; 179711; 179712; 179725; 179726; 179727; 179728; 179729; 179730; 179731; 179732; 179733; 179734)  Mitomycin C (384925; 384926; 384927; 384928; 384929)  Mitozantrone (382125; 382126; 382127; 382128)  Mycophenolate mofetil (103401; 103425; 103426; 103427)  Nilotinib (403825; 403826)  Nivolumab (406925; 406926; 406927)  Obinutuzumab (407825; 407826)  Octreotide (251101; 251102; 251103; 251104; 251105; 251106; 251126; 251127; 251128; 251129; 251130)  Octreotide (somatostatin analogue) (251201; 251202; 251203; 251204; 251205; 251206)  Octreotide LAR (somatostatin analogue) (251125; 251225; 251226; 251227)  Olaparib (251101; 251102; 251103; 251104; 251105; 251106; 251126; 251127; 251128; 251129; 251130)  Omalizumab (403925; 403926)  Oxaliplatin (383225; 383226; 383227; 383228; 383229)  Paclitaxel (381525; 381526; 381527; 381528; 381529; 381530)  Palbociclib (412525; 412526; 412527)  Pazopanib (394325; 394326)  Pegaspargase (400525; 400526)  Pembrolizumab (407225; 407226; 407227)  Pemetrexed (408825; 408826; 408827)  Pentostatin [Deoxycoformycin] (385025)  Pertuzumab (407725; 407726; 407727)  Procarbazine hydrochloride (204701)  Rituximab [Mabthera] (381725; 381726; 381727)  Rituximab [Riximyo] (412425; 412426; 412427)  Ruxolitinib (410625; 410626; 410627)  Secukinumab (410725)  Siltuximab (406425; 406426)  Sirolimus (376925; 376926; 386925; 386926; 386927)  Stilboestrol (218301)  Sunitinib (392125; 392126; 392127)  Tacrolimus (108801; 108802; 108825; 108826)  Tamoxifen citrate (221801; 221802)  Temozolomide (373325; 373326; 373327; 385625; 385626; 385627; 385628; 385629; 385630)  Teniposide (385125; 385126)  Thalidomide (384525; 384526)  Thioguanine (225201)  Thiotepa (225701; 225725)  Tocilizumab (411525; 411526; 411527; 411528)  Toremifene (371525)  Trastuzumab (381825; 381826; 381827)  Trastuzumab emtansine (412225; 412226; 412227)  Tretinoin (228625)  Venetoclax (412025; 412026; 412027; 412028)  Vinblastine sulphate (231901; 231925)  Vincristine sulphate (232001; 232002; 232025)  Vinorelbine (381625; 381626; 381627) | |

**Table S5: List of comorbidities**

| **ICD-10-AM-iii Code** | **Code Description** |
| --- | --- |
| J40-J44 | Chronic obstructive pulmonary disease (COPD) |
| E10-E14 | Diabetes mellitus |
| M10 | Gout |
| I60-I69 | Cerebrovascular disease (including stroke) |
| I20-I25 | Ischaemic heart disease (including acute myocardial infraction) |
| N18 | Chronic kidney disease |
| K700-K709, K721-K746 | Liver disease |
| MO05-M06 | Seropositive and other rheumatoid arthritis |
| M07 | Psoriatic and enteropathic arthropathies |
| M32 | Systemic lupus erythematosus |
| K580 | Irritable bowel syndrome |
| L40 | Psoriasis |
| M450 | Ankylosing spondylitis |

**Table S6: Baseline Characteristics of Study Cohorts by Herpes Zoster Vaccination Status (before cleaning)**

| **Characteristics** | | **Total**  **N= 835125** | | **Vaccinated (%)**  **N = 278375 (33.33%)** | | **Unvaccinated (%)**  **N= 556750 (66.66%)** | | **p-value** |
| --- | --- | --- | --- | --- | --- | --- | --- | --- |
| Age | 45 – 49 | 123 | 0.01% | 41 | 0.01% | 82 | 0.01% | **1.0** |
|  | 50 – 54 | 468 | 0.06% | 156 | 0.06% | 312 | 0.06% | **1.0** |
|  | 55 – 59 | 768 | 0.09% | 256 | 0.09% | 512 | 0.09% | **1.0** |
|  | 60 – 64 | 69,561 | 8.33% | 23,187 | 8.33% | 46374 | 8.33% | **1.0** |
|  | 65 – 69 | 280,554 | 33.59% | 93,518 | 33.59% | 187036 | 33.59% | **1.0** |
|  | 70 – 74 | 257,490 | 30.83% | 85,830 | 30.83% | 171660 | 30.83% | **1.0** |
|  | 75 – 76 | 183,318 | 21.95% | 61,106 | 21.95% | 122212 | 21.95% | **1.0** |
|  | ≥ 80 | 42,843 | 5.13% | 14,281 | 5.13% | 28562 | 5.13% | **1.0** |
| **Sex** | Male | 399,045 | 47.78% | 133,015 | 47.78% | 266,030 | 47.78% | **1.0** |
|  | Female | 436,080 | 52.23% | 145,360 | 52.22% | 290,720 | 52.22% | **1.0** |
| Ethnicity  (Level 1 ethnic codes) | Māori (2) | 48263 | 5.78 | 16,081 | 5.78% | 32,182 | 5.78% | **1.0** |
|  | Pacific Peoples (3) | 27773 | 3.33% | 9,170 | 3.29% | 18,603 | 3.34% | **1.0** |
|  | European (1) | 669369 | 80.15 | 225,707 | 81.08% | 443,662 | 79.69% | **1.0** |
|  | Asian (4) | 72438 | 8.67% | 24,129 | 8.67% | 48,309 | 8.68% | **1.0** |
|  | MELAA (5) | 3767 | 0.45% | 1,222 | 0.44% | 2,545 | 0.46% | **1.0** |
|  | Other Ethnicity (6) | 1551 | 0.19% | 515 | 0.19% | 1,036 | 0.19% | **1.0** |
|  | Residual Categories (9) | 11964 | 1.43% | 1,551 | 0.56% | 10,413 | 1.87% | **1.0** |
| **NZDep2013**  **(Quintiles)** | Quin 1 | 167,587 | 20.06% | 58,924 | 21.17% | 108,663 | 19.52% | **0.98** |
|  | Quin 2 | 160,971 | 19.28% | 55,549 | 19.95% | 105,422 | 18.94% | **1.0** |
|  | Quin 3 | 170,719 | 20.44% | 57,738 | 20.74% | 112,981 | 20.29% | **1.0** |
|  | Quin 4 | 176,281 | 21.11% | 58,194 | 20.90% | 118,087 | 21.21% | **1.0** |
|  | Quin 5 | 159,567 | 19.11% | 47,970 | 17.23% | 111,597 | 20.04% | **1.0** |
| **Immune suppression** | Yes | 55,752 | 6.68% | 16,964 | 6.09% | 38,788 | 6.97% | **0.99** |
|  | No | 779,373 | 93.32% | 261,411 | 93.91% | 517,962 | 93.03% | **1.0** |
| **COPD** | Yes | 22,606 | 2.71% | 6,748 | 2.42% | 15,858 | 2.85% | **1.0** |
|  | No | 812,519 | 97.29% | 271,627 | 97.58% | 540,892 | 97.15% | **1.0** |
| **DM** | Yes | 17,880 | 2.14% | 6,073 | 2.18% | 11,807 | 2.12% | **0.96** |
|  | No | 817,245 | 97.86% | 272,302 | 97.82% | 544,943 | 97.88% | **0.98** |
| **Kidney disease** | Yes | 2,276 | 0.27% | 432 | 0.16% | 1,844 | 0.33% | **0.98** |
|  | No | 832,849 | 99.73% | 277,943 | 99.84% | 554,906 | 99.67% | **1.0** |
| **Liver disease** | Yes | 743 | 0.09% | 122 | 0.04% | 621 | 0.11% | **1.0** |
|  | No | 834,382 | 99.91% | 278,253 | 99.96% | 556,129 | 99.86% | **1.0** |
| **IHD** | Yes | 68,922 | 8.25% | 27,975 | 10.05% | 40,947 | 7.35% | **0.99** |
|  | No | 766,203 | 91.75% | 250,400 | 89.95% | 515,803 | 92.65% | **1.0** |
| CVD | Yes | 32,253 | 3.86% | 11,047 | 3.97% | 535,544 | 3.81% | **1.0** |
|  | No | 802,872 | 96.14 | 267,328 | 96.03% | 21,206 | 96.16% | **1.0** |
| **Gout** | Yes | 4,290 | 0.51% | 1,501 | 0.54% | 2,789 | 0.50% | **1.0** |
|  | No | 830,835 | 99.49% | 276,874 | 99.46% | 553,961 | 99.50% | **1.0** |
| **AI disease** | Yes | 2,771 | 0.33% | 579 | 0.21% | 2,192 | 0.39% | **1.0** |
|  | No | 832,354 | 99.67% | 277,796 | 99.79% | 554,558 | 99.61% | **1.0** |
| **Cancer** | Yes | 128,860 | 15.43% | 41,702 | 14.98% | 87,158 | 15.65% | **1.0** |
|  | No | 706,265 | 84.57% | 236,673 | 85.02% | 469,592 | 84.35% | **1.0** |

NZDep2013: Index of deprivation 13 divided into five quintiles; DM: Diabetes mellitus; COPDb: Chronic obstructive pulmonary disease, IHDc: Ischaemic heart disease; CVS: Cerebrovascular diseases including stroke, AI diseases: Autoimmune diseases (Seropositive and other rheumatoid arthritis, psoriatic and enteropathic arthropathies, systemic lupus erythematosus, irritable bowel syndrome, psoriasis, ankylosing spondylitis; DHB: District Health Board

p-value, comparing the proportions of baseline characteristics before and after cleaning.

MELAA: Middle Eastern / Latin American / African

**Table S7: Distribution of herpes zoster cases by calendar time (2018 – 2021)**

| **Event Period** | **Community Zoster** | | | **Hospitalised herpes zoster** | **Hospitalised postherpetic neuralgia** |
| --- | --- | --- | --- | --- | --- |
|  | **Unvaccinated** | **Vaccinated** | **Total** | **Total** | **Total** |
| 2018-03 | 344 | 0 | 344 | 19 | ≤5 |
| 2018-04 | 295 | 0 | 295 | 27 | 14 |
| 2018-05 | 290 | ≤ 5 | 293 | 20 | ≤5 |
| 2018-06 | 217 | 14 | 231 | 15 | 6 |
| 2018-07 | 239 | 23 | 262 | 12 | ≤5 |
| 2018-08 | 250 | 33 | 283 | 28 | ≤5 |
| 2018-09 | 196 | 19 | 215 | 20 | ≤5 |
| 2018-10 | 196 | 30 | 226 | 15 | 8 |
| 2018-11 | 209 | 34 | 242 | 21 | ≤5 |
| 2018-12 | 207 | 31 | 238 | 24 | 8 |
| 2019-01 | 181 | 45 | 226 | 23 | ≤5 |
| 2019-02 | 170 | 35 | 205 | 15 | ≤5 |
| 2019-03 | 180 | 42 | 222 | 22 | ≤5 |
| 2019-04 | 163 | 36 | 199 | 16 | ≤5 |
| 2019-05 | 171 | 38 | 209 | 24 | 6 |
| 2019-06 | 137 | 18 | 155 | 18 | 6 |
| 2019-07 | 147 | 43 | 190 | 21 | ≤5 |
| 2019-08 | 145 | 53 | 198 | 14 | ≤5 |
| 2019-09 | 131 | 46 | 177 | 13 | ≤5 |
| 2019-10 | 138 | 48 | 186 | 19 | ≤5 |
| 2019-11 | 118 | 39 | 157 | 15 | ≤5 |
| 2019-12 | 124 | 46 | 170 | 20 | 10 |
| 2020-01 | 130 | 47 | 177 | 22 | 7 |
| 2020-02 | 129 | 44 | 173 | 19 | ≤5 |
| 2020-03 | 133 | 48 | 181 | 16 | ≤5 |
| 2020-04 | 94 | 35 | 129 | 18 | ≤5 |
| 2020-05 | 88 | 42 | 130 | 14 | 6 |
| 2020-06 | 90 | 49 | 139 | 15 | ≤5 |
| 2020-07 | 108 | 43 | 151 | 20 | ≤5 |
| 2020-08 | 122 | 48 | 170 | 25 | ≤5 |
| 2020-09 | 91 | 55 | 146 | 17 | ≤5 |
| 2020-10 | 94 | 36 | 130 | 20 | 6 |
| 2020-11 | 79 | 46 | 125 | 11 | ≤5 |
| 2020-12 | 103 | 52 | 155 | 21 | ≤5 |
| 2021-01 | 82 | 33 | 115 | 18 | ≤5 |
| 2021-02 | 77 | 41 | 118 | 20 | ≤5 |
| 2021-03 | 88 | 51 | 139 | 26 | ≤5 |

**
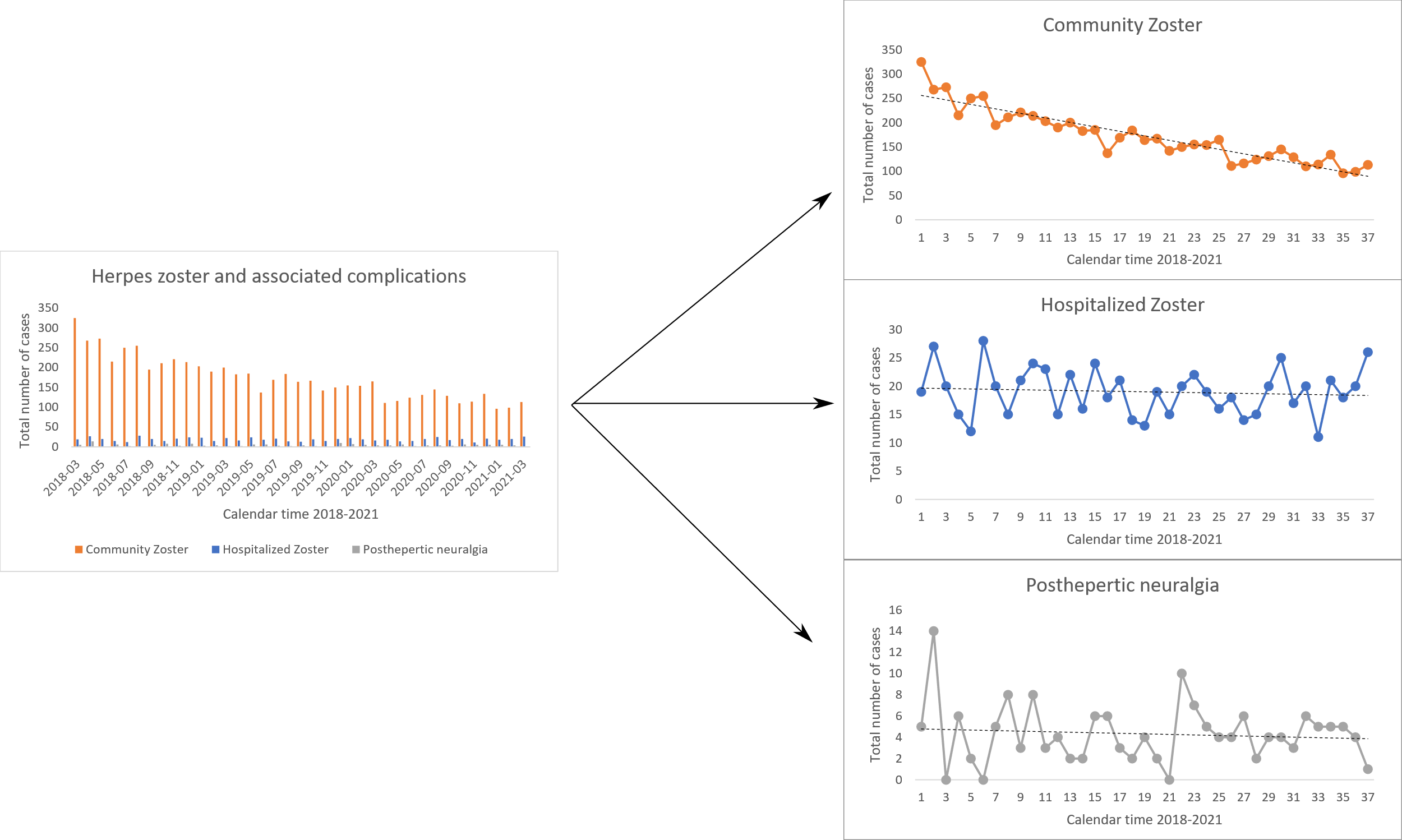
**

**Figure S4: Distribution of herpes zoster and associated complications by calendar time (2018 – 2021)**
